# Family caregivers’ preparedness to support the physical activity of patients at risk for hospital readmission in rural communities: an interpretive descriptive study

**DOI:** 10.1186/s12913-022-08289-4

**Published:** 2022-07-13

**Authors:** Mary T. Fox, Jeffrey I. Butler, Souraya Sidani, An Nguyen

**Affiliations:** 1 School of Nursing, York University Centre for Aging Research and Education, Faculty of Health, York University, HNES suite 343, 4700 Keele Street, Toronto, Ontario M3J 1P3 Canada; 2 Faculty of Community Services, Ryerson University, 350 Victoria Street, Toronto, ON M5B 2K3 Canada; 3 School of Nursing, Faculty of Health, York University, 4700 Keele Street, Toronto, Ontario M3J 1P3 Canada

**Keywords:** Physical activity, Families, Caregivers, Preparedness, Rural healthcare, Hospital readmission, Hospital-to-home transitional care

## Abstract

**Background:**

Physical activity in the post-discharge period is important to maximize patient recovery and prevent hospital readmission. Healthcare providers have identified family caregivers as potential facilitators of patients’ engagement in physical activity. Yet, there is very little research on family caregivers’ perspectives on their preparedness to support the physical activity of patients, particularly those at risk for hospital readmission in rural communities. Accordingly, this study explored the challenges related to family caregivers’ preparedness to support the physical activity of a recently discharged, rural-dwelling relative at risk for hospital readmission.

**Methods:**

In this interpretive descriptive study, semi-structured interviews were conducted by telephone with 16 family caregivers. Interview transcripts were analyzed using thematic analysis.

**Results:**

Participants were predominantly women (*n* = 14; 87.5%) with an average age of 49 years (range 26–67) who were the primary caregivers of a relative who had been hospitalized for a medical illness (*n* = 12; 75%) and was at high risk for hospital readmission. Four themes were identified: 1) family caregivers generally felt unprepared to support their relative’s physical activity, 2) some family caregivers believed that rest was more important than physical activity to their relative’s recovery, 3) insufficient physical activity preparation led to family caregiver-relative conflicts, and 4) to defuse these conflicts, some family caregivers wanted healthcare providers to be responsible for promoting physical activity.

**Conclusions:**

Despite assertions that family caregivers are a potential source of support for patient physical activity, our findings indicate that family caregivers are largely unprepared to assume that role and that more work needs to be done to ensure they can do so effectively. We suggest that healthcare providers be conscious of the potential for family caregiver-patient conflict surrounding physical activity, assess family caregivers’ ability and willingness to support physical activity, educate them on the hazards of inactivity, and provide physical activity instructions to family caregivers and patients conjointly. Preparing family caregivers to support their relative’s physical activity is particularly important given the current emphasis on early discharge in many jurisdictions, and the limited formal healthcare services available in rural communities.

**Supplementary Information:**

The online version contains supplementary material available at 10.1186/s12913-022-08289-4.

## Background

Physical activity refers to any bodily movement that expends energy and is not limited to exercise but also includes movements such as walking and doing housework [1]. Low physical activity in the post-discharge period is associated with poor health outcomes and 30-day hospital readmission [2–4]. Accordingly, promoting patients’ physical activity in the post-discharge period is important to maximize their recovery and prevent hospital readmissions.

Family caregivers (FCs) are often responsible for supporting their relative’s recovery after hospital discharge. While healthcare providers have identified FCs as potential facilitators of patients’ engagement in physical activity [5, 6], very few studies have examined the challenges related to FCs’ preparedness to support patients’ physical activity, and those that did were mainly conducted in the hospital setting – limiting their applicability to the post-discharge period. For example, in qualitative interviews with FCs of hospitalized patients, Lim and colleagues found that FCs recognized the need for their relative to mobilize but feared their relative might fall [7]. Similarly, in a survey of FCs, Najjar’s team found that FCs believed that their relative should regularly ambulate while in hospital but were concerned about the potential dangers of doing so [8]. We found only one study exploring FCs’ perspectives of their preparedness after hospital discharge; in that study, FCs reported feeling prepared to manage their relative’s physical functional needs but the study interviewed FCs 6 months following their relative’s hospital discharge, did not focus on FCs’ perspectives of supporting physical activity in the post discharge period, and all participants were receiving home care services before and following hospital discharge [9]; the transferability and relevance of the findings to FCs’ preparedness to support the physical activity of a recently hospitalized relative are thus questionable.

Furthermore, no studies have focused specifically on FCs of patients at risk for hospital readmission in rural communities. This is a crucial gap because nowhere are FCs more integral to patients’ physical activity following hospitalization than in rural communities. Rural communities have limited healthcare resources and face barriers to healthcare access because of distance, resulting in many patients relying heavily on their FCs [10].

Rural communities also typically have higher proportions of older people and people with multiple chronic conditions [10], which are risk factors for physical inactivity [11] and hospital readmission [2]. Understanding FCs’ perceived preparedness to support physical activity is crucial to knowing how to best position FCs to assist their relative after discharge. Consequently, this study explored the challenges related to FCs’ preparedness to support the physical activity of a recently hospitalized, rural-dwelling relative at risk for readmission.

## Methods

### Study design

This qualitative study used an interpretive descriptive approach, which guides researchers in developing an interpretive understanding of a health or clinical experience with the potential to inform practice [12]. This study was part of a larger multi-method project in which FCs were interviewed on their preparedness to support multiple aspects of a relative’s post-discharge care [13, 14].

### Ethical considerations

Ethics approval was obtained from the Office of Research Ethics at York University, Certificate#: e2018–014, and from the Research Ethics Office at Health Sciences North, Project# 18–053. Written informed consent was obtained from all participants. All methods were carried out in accordance with the relevant guidelines and regulations of the World Health Organization’s Declaration of Helsinki.

### Sample and setting

Our purposeful sample consisted of adults aged 18+ who were the unpaid, primary caregiver of an adult relative discharged home to a rural community following hospitalization for a medical illness or surgical procedure, able to stand or weight bear, and at risk for hospital readmission. FCs were recruited through hospital staff referrals as well as study flyers circulated throughout rural Southwestern and Northeastern Ontario. We targeted 8 to 12 FCs per region (for a total of 16 to 24 FCs) until informational saturation (the point at which no new codes or categories emerged) was reached. Two research team members (MF and JB) separately ascertained that saturation, which was examined throughout data collection, had been achieved with 16 FCs.

### Data collection

Data were collected in 2018 and 2019. FCs were screened for eligibility, and information on their sociodemographic characteristics was obtained using the measures reported below. Qualitative interviews were guided by a semi-structured interview guide to collect data on FCs’ perceived preparedness to support their relative’s physical activity. A doctoral trained Research Associate (RA) conducted the one-on-one telephone interviews with FCs. The RA (JB), a man with more than fifteen years of related experience in qualitative research, explained to FCs that he was a sociologist and the study RA with an interest in learning more about their recent hospital and post-discharge experience. The other research team members are women with backgrounds in nursing; two are tenured faculty (MF and SS) and one is a doctoral student (AN) focusing her studies on FC support of patient physical functioning following hospital discharge. The lead author (MF) has extensive experience studying physical functioning surrounding the hospitalization period. No research team member had a prior relationship with the participants.

Participants were asked to be in a quiet environment free of distractions during the interview [13]. The interviews took about one hour and were audio-recorded and transcribed. Field notes were taken following data collection which was completed within 30 days after FCs’ relatives’ hospital discharge. To lessen burden, transcripts and findings were not returned to participants for verification.

#### Screening and sociodemographic measures

To determine if potential participants were caring for a relative at risk for hospital readmission, the LACE index was employed. The LACE uses four variables to assess risk for hospital readmission during the 30-day post-discharge period: length of stay in hospital (“L”), acuity of hospital admission (“A”), comorbidities (“C”), and ED visits in the six months prior to admission (“E”) [15]. LACE data were obtained through self-report or from medical records. LACE indices from 5 to 9 indicate moderate risk of hospital readmission whereas indices >10 indicate high risk [15]. The LACE index was previously validated as a predictor of 30-day hospital readmission and demonstrated high discriminant ability [16].

To establish that potential participants were caring for a relative living in a rural community, we used the Rurality Index of Ontario – a census-based metric that uses population size and travel time to the nearest healthcare center. Scores were acquired by inputting the relative’s postal code into an online calculator; indices > 40 indicate rural residency, with higher indices indicating higher rurality [17]. Other eligibility criteria (e.g., age) were screened with self-report measures.

We used the corresponding item of the Basic Physical Capability Scale [18] to determine if potential participants’ relatives were able to stand or weight bear on discharge (indicated by a score of 1). Data were obtained by FC report. The scale demonstrated construct validity (all items fit the Rasch model testing) and internal consistency reliability (Cronbach’s alpha = 0.79) [18].

Additional FC eligibility criteria included that participants were age 18+, English speaking and reading, and a primary unpaid caregiver. These criteria were screened with self-report measures. FCs’ sociodemographic profile (highest level of education, employment status, and household income) were assessed using standard self-report questions.

#### Qualitative interviews

Interviews were conducted using a semi-structured interview guide (Appendix A) which had been pilot tested. Participants were invited to reflect on their recent hospital and post-discharge experience and to describe any parts of their relative’s care at home for which they felt unprepared, as well as any help they received at the hospital or at home to prepare them to support their relative’s physical activity. FCs were also asked to discuss if/how they could have been better prepared to support their relative’s physical activity. To prompt participants to clarify or expand on their responses, FCs were asked if there was anything specific about the physical activity recommendations (if any had been provided) they found difficult to implement and what would have made it easier for them to support their relative’s physical activity at home.

### Data analysis

Participants’ average standing on the screening and sociodemographic measures was summarized with descriptive statistics using SPSS. Qualitative data analysis, facilitated by N-Vivo, was done concurrently with data collection. We used inductive thematic analysis, which entailed creating preliminary codes and grouping them into hierarchically organized meta- and sub-categories [19]. One of the coding authors (JB) reviewed the audio recordings and both (JB and MF) reviewed the transcripts and codes. Both coding authors (JB and MF) first coded and analyzed the data independently. Definitions for each code, category, and subcategory were developed, their interconnections recorded, and exemplars for each were identified. Salient categories were refined into overarching, interpretive themes. As themes were refined, any areas of disagreement of the coding authors were discussed until intersubjective consensus was reached. MF brought her viewpoint as a nurse and JB as a sociologist to the discussion.

Where relevant, we accounted for negative cases and trends in participants’ narratives based on demographics (e.g., gender). Strategies for trustworthiness were employed throughout the study [20]. Credibility was ensured through prolonged, iterative engagement with the narrative data, and independent analysis by two team members. Dependability was achieved by in-depth description of the methods and having all team members review the findings. Transferability was ensured through the provision of data on participant and setting characteristics [20]. Confirmability was ensured by using a semi-structured interview guide that allowed participants to articulate their experiences in their own terms, documenting an audit trail and field notes immediately following interviews, and including verbatim quotes in the findings.

## Results

A total of 56 FCs expressed interest in the study but 6 declined the offer to participate after hearing more about what it entailed, and 22 were ineligible. Of the 28 eligible, consenting FCs, two became ineligible when their relative’s status changed, and 10 withdrew (most implicitly, by not responding to requests to schedule an interview). The final sample size was 16.

Individual FC characteristics are outlined in Table 1. Most FCs were women (*n* = 14; 87.5%) with an average age of 49 years (range 26–67). All self-identified as Caucasian. The majority were married or common law (*n* = 11; 68%). Most had a college diploma, apprenticeship, or trades certificate (*n* = 10; 62.5%), were employed full-time (*n* = 10; 62.5%) and had an annual household income less than $70,000 before taxes (*n* = 9; 56.3%).

**Table 1:** Individual level family caregiver characteristics

Participant	Gender	Marital Status	Age	Education	Household Income	Primary Caregiver to …	Employment Status outside of home	Lives with relative	Age of relative	Reason for relative’s hospital admission	RIO score of relative	LACE score of relative
FC1	Woman	Single	55	High school diploma or equivalent	$40,000-49,999	Mother	Full-time	No	82	Medical	79	14
FC2	Woman	Married	49	Bachelor’s degree	$70,000+	Mother	Full-time	No	87	Medical	41	8
FC3	Woman	Divorced	54	College diploma, trades certificate or apprenticeship	$50,000-59,999	Mother	Full-time	No	82	Surgical	51	13
FC4	Woman	Married	59	College diploma, trades certificate or apprenticeship	$30,000–$39,999	Husband	Unemployed	Yes	70	Medical	46	15
FC5	Woman	Common law	43	College diploma, trades certificate or apprenticeship	$40,000-49,999	Father	Full-time	No	68	Surgical	69	8
FC6	Woman	Married	49	High school diploma or equivalent	Less than $10,000	Mother	Unemployed	Yes	87	Medical	89	13
FC7	Woman	Common law	26	College diploma, trades certificate or apprenticeship	$70,000+	Father	Full-time	No	61	Surgical	51	8
FC8	Man	Single	47	Some high school	$50,000-59,999	Mother	Unemployed	Yes	72	Medical	79	11
FC9	Woman	Married	45	College diploma, trades certificate or apprenticeship	$70,000+	Son	Full-time	Yes	20	Medical	42	14
FC10	Woman	Married	67	College diploma, trades certificate or apprenticeship	$70,000+	Husband	Full-time	Yes	73	Medical	52	8
FC11	Woman	Married	34	College diploma, trades certificate or apprenticeship	$30,000–$39,999	Husband	Part-time	Yes	48	Medical	50	11
FC12	Woman	Married	63	College diploma, trades certificate or apprenticeship	$60,000-69,999	Husband	Part-time	Yes	63	Medical	50	15
FC13	Woman	Single	60	High school diploma or equivalent	$70,000+	Father	Retired	No	86	Medical	50	9
FC14	Woman	Single	46	Master’s degree	$60,000-69,999	Mother	Full-time	Yes	67	Medical	48	9
FC15	Man	Married	29	College diploma, trades certificate or apprenticeship	$70,000+	Wife	Full-time	Yes	27	Medical	50	8
FC16	Woman	Married	54	College diploma, trades certificate or apprenticeship	$70,000+	Husband	Full-time	Yes	54	Surgical	50	11

Note: RIO Rurality Index of Ontario, LACE length of stay (L), acuity of the admission (A), comorbidity of the patient (C) and emergency department use in the duration of 6 months before admission

FC family caregiver. Relative refers to family caregiver’s relative

Most FCs were the primary caregiver of a parent (*n* = 9; 56.3%) and were living in the same home as their relative (*n* = 10; 62.5%) in a community with a median rurality index of 50 (range 41–89). Most FCs were the primary caregiver of a relative who had been hospitalized for a medical illness (*n* = 12; 75%) and was at high risk for hospital readmission, manifested by a mean LACE index of 10.9 (+ 2.7).

Qualitative analysis revealed four themes: 1) FCs generally felt unprepared to support their relative’s physical activity, 2) some FCs believed that rest was more important than physical activity to their relative’s recovery, 3) insufficient physical activity preparation led to FC-relative conflicts, and 4) to defuse these conflicts, some FCs wanted healthcare providers to be responsible for promoting physical activity.

### Theme 1: family caregivers generally felt unprepared to support their relative’s physical activity

FCs described receiving very limited preparation in hospital or at home on how to support physical activity; only one FC reported having received sufficient preparation in the form of “very specific instructions about how much he [FC5’s father] should be doing, his leg flexes, and arm lifts.” Most expressed having received no information at all. For example, FC7 noted that she “never was spoken to at all about his [her father’s] condition by anybody”, and FC8 similarly recalled that “I’ve never talked to anybody. I’ve never had anybody talk to us [FC and his mother], it’s, ‘Oh, you’re good to go’ [to be discharged].”

Specifically, FCs emphasized that the preparation they and their relative had received was inadequate in three ways. First, those FCs who did receive information from healthcare providers characterized it as vague and insufficiently detailed to guide FCs on how to support their relative’s physical activity. For example, some FCs explained that healthcare providers’ instructions included “get as much activity as you can”, “walk but don’t overdo it”, and “just take it easy.”

Second, FCs received insufficient guidance on what to expect in terms of their relative’s capacity for physical activity after discharge. In other words, FCs were unsure what their relative’s starting point for returning to their pre-hospitalization baseline would be. FC7, for instance, criticized that she did not even “know if he [her father] was going to be able to get in and out of bed by himself.”

Third, FCs received insufficient guidance on the progression of their relative’s recovery and how physical activity figured into it. They highlighted numerous ways in which they were uncertain about the trajectory it would take, and how they could support it. For example, FCs such as FC15, recounted not knowing what level of physical activity their relative should engage in at a given point in the recovery process:

I think what we [FC15 and his wife] were missing was a clear progression, path, what to expect, how many days in terms of mobility and getting around and when we need to start progressing it. Not having any clear, again plan of how much can she [FC15’s wife] move on the first day and how much can she move on the second and third day [after coming home from hospital] made it very difficult, and has still made it difficult, trying to figure out how much is too much and is this amount of pain just a normal part of recovery or has it now hit the point where we’re pushing too hard [physically] and going the opposite way from recovery by causing too much strain.

Consequently, FCs were unsure how to best support their relative’s physical activity after discharge; that is, they lacked information on the concrete steps and physical activities they needed to encourage their relative to engage in to facilitate recovery. FC7, for instance, recalled telling her father that “I don’t even really know what’s best for you to get up the stairs at the front of the house. Should you be leading with your dominant foot? Or should you be leading with your injured foot?” FC7 recalled needing to know more about the “simple things, just day to day, you know, how long he should be up walking for?” Typically, FCs were only confronted with the full ramifications of their lack of preparation when their relative returned home from hospital and realized that they did not know how to support their relative’s physical activity. FCs acknowledged the importance of their relative’s return to physical activity, but conceded that, as FCs, they were unprepared for how to help their relative achieve that goal.

### Theme 2: some family caregivers believed that rest was more important than physical activity to their relative’s recovery

In the absence of direction explicitly outlining how to support their relative’s physical activity, FCs relied on their “common sense”, which was informed by the belief that rest promotes healing, and too much physical activity threatens recovery. In practice, that meant emphasizing rest over physical activity. FC7 for instance, explained that when she “didn’t get anything [physical activity plan or instructions] from them [healthcare providers]”, she automatically assumed “he’s [FC7’s father] supposed to get lots of rest … just came from common sense, make sure he’s getting enough rest.” She went on to describe how she saw her father’s eagerness to resume physical activity as something to be limited:

You need to ensure that your body has that time to heal. I know a lot of people go home after surgery and want to lay in bed. It was kind of the opposite for my dad. [He] wanted to get up and get moving and get back to his daily activities. And that's the goal, but, I think you can’t go at 100 percent all the time while you’re healing.

FCs’ entrenched views on the importance of rest were evidenced by their criticism of healthcare providers who, FCs believed, pushed their relative too hard after a period of prolonged inactivity during hospitalization. For some FCs, the belief that rest had to be promoted was so strong that they disputed healthcare providers’ instructions that heavily emphasized physical activity. FC7 noted with frustration that “they told him [FC7’s father], ‘You can’t walk enough.’ Well, I would argue that [it was too much] when he was out walking for five hours, up and down hills and trying to help with projects around the house. And then the next day, he felt like crap all day.”

FC2 also recounted resisting healthcare providers’ guidance to let her mother do as much for herself as possible. As FC2 put it: “She [FC2’s mother] can do it, but the thing is, if she’s coming home and she’s supposed to recover, she shouldn’t be doing those things. She will say that she can do all those things, clean, cook, laundry, but the thing is she shouldn’t be [doing them]. And, I see that [she can do it herself], but then I do it for her, just to help out so that she can recover, you know, get some rest.”

### Theme 3: insufficient physical activity preparation led to family caregiver-relative conflicts

For those FCs who attempted to support physical activity, unclear guidelines created space for their relative to challenge their attempts. These FCs explained how, when they encouraged their relative to be more physically active, they experienced pushback. Notably, gender appeared to figure prominently in these dynamics; all the FCs who flagged their relative’s resistance to physical activity as problematic were women (i.e., wives/partners or daughters) caring for a male relative.

Analysis identified that FCs perceived pushback from their relatives as taking two main forms. First, some FCs reported that their relative actively questioned their credibility when it came to promoting physical activity and strategies to support it. As one FC explained, her father “thinks we [family] don’t know what we’re talking about.” Other FCs recounted how their interpretation of healthcare providers’ instructions was viewed as suspect by their relative, who was skeptical that their FC had fully understood the instructions.

Second, some FCs described their relative’s outright refusal to engage in physical activity. For example, FC13 noted:

That’s always a challenge - to get him [her father] to move … When we try to get him out walking, he just is very stubborn and won’t go. Or he refuses to do it. We’ve tried since he’s been home to get him out walking, and he just refuses to go.

FC4 concurred that the greatest “difficulty I had was to get him [FC4’s husband] to do it [walk]”, because he made clear that “I don’t have to listen to you.” FC4 suggested that this friction was the result of the established power dynamics of their relationship being upset by her husband’s illness. As FC4 explained, “it’s kind of like a child with their parent, you know? Just the whole resistance part.” She maintained that, because her husband felt disempowered, he was using interactions surrounding physical activity as an opportunity to assert himself. She elaborated that “it’s a control thing, because he doesn’t have a lot of control over some things in his life, but he can control whether or not I help him, control him. If he’ll think I’m trying to control him he doesn’t want that, he wants his own control, autonomy.” Other FCs were less empathetic, and when faced with resistance from their relative they responded by relentlessly policing their relative’s physical activity. These FCs insisted that promoting physical activity requires taking on an authoritative, taskmaster role to ensure their relative engages in sufficient physical activity.

### Theme 4: to defuse these conflicts, some FCs wanted healthcare providers to be responsible for promoting physical activity

With such conflict in mind, some FCs indicated that, ideally, it would be healthcare providers’ responsibility to support their relative’s physical activity because a healthcare provider’s credibility and authority were more likely to be accepted. FC6 noted that:

I want her [FC6’s mother] to do more but there's nothing more I can do for her here by myself. She sees physiotherapy with me as just a game. When she does it with the physiotherapist, she understands it's serious and she needs to do it.

Accordingly, some FCs believed a healthcare provider would help their relative be more physically active. When asked about strategies to help their relative participate in physical activity, FC4 explained that “outside motivation … is something that works better with him [FC4’s husband]. For his PSW [personal support worker], he will do things for her [that he won’t do for me].” As FC10 conveyed “it’s always different when somebody else says it, right? Like the ones closest to you are the ones you least listen to.” FC13 similarly noted that:

I think having somebody else involved in telling him [FC13’s father] to get up and move a little bit more, I think that would be very helpful … they could kind of reiterate that it’ll only get better if you keep moving … getting it from somebody else other than his kids. I think he’d listen to somebody else.

### Relationship between themes

Our themes illustrate that FCs’ unpreparedness to support patient physical activity spurs them to rely on their own common sense, sparks interpersonal conflict, and leads to the shirking of responsibility for supporting patient physical activity. The themes thus account for both the external and internal constraints on FCs’ ability to support their relative’s physical activity. On the one hand, our first theme, i.e., insufficient preparation by healthcare providers, captures a key external constraint beyond FCs’ control which leaves them ill-informed and poorly positioned to help their relative be physically active. On the other hand, our second, third, and fourth themes describe how the absence of guidance on physical activity creates internal constraints for FCs. Specifically, FCs rely on their common sense which prioritizes rest over physical activity. In addition, they want to avoid interpersonal conflict. Lastly, they would prefer to deflect the role of promoting physical activity onto healthcare providers. Taken together, these constraints undermine FCs’ ability to support physical activity and illustrate the complex, interrelated factors influencing FCs’ ability to support their relative’s physical activity in the post-discharge period. In short, FCs’ reliance on their own judgement, FC-patient conflict, FCs’ unwillingness to support physical activity – manifested in their shirking of responsibility for physical activity – are outcomes of their unpreparedness.

## Discussion

Our study adds to the sparse literature on the challenges related to FCs’ preparedness to support physical activity in the post-discharge period and stakes out important new ground by charting this issue specifically in the rural context. Our findings are embedded in rural communities, which are typified by older age and higher levels of chronicity [10], but less access to the healthcare system that their urban counterparts [21]. Consequently, FCs play an outsized role in rural healthcare. It is thus disconcerting that they are ill-positioned to support the physical activity of patients at risk for hospital readmission.

In general, we identified that FCs of predominantly medical patients at high risk for hospital readmission do not feel prepared. This finding differs from Chase et al.’s study in which FCs expressed feeling well prepared to manage their relative’s physical functioning [9]. Our study’s timely addition to this literature is essential because, even though current guidelines stress the importance of promoting physical activity during and following hospitalization, evidence indicates that many patients continue to have limited physical activity during hospitalization and consequently leave hospital with newly acquired declines in their physical functioning [22]. As a result, preparing FCs in how to support physical activity after hospital discharge is crucial to patients’ ability to return to their pre-hospital baseline levels of physical activity which, in turn, can influence recovery and sustainable hospital-to-home transitions.

That FCs received insufficient guidance on their relative’s capacity for physical activity and how to support it parallels Najjar et al.’s findings that few FCs receive information on how to support their relative’s physical activity; FCs in that study indicated that their lack of knowledge was the greatest barrier in assisting their hospitalized relative to mobilize [8]. Our finding that FCs needed more information on the trajectory of their relative’s recovery, and how physical activity figures into it, parallels other studies concluding that FCs need more information on their relative’s health trajectory [23], what to expect at home [24], and how to support patients’ physical functional needs after discharge [9]. In Mitchell et al.’s study, for instance, FCs identified the importance of having detailed post-discharge instructions to enhance their preparedness [25]. Crucially, FCs in that study maintained that, when they were prepared, they felt more competent, confident, and better able to adhere to post-discharge instructions [25].

Regarding our finding that FCs’ beliefs shape how they support physical activity, we were only able to locate a few relevant studies. Our finding that, in the absence of direction explicitly outlining how to support their relative’s physical activity, FCs relied on “common sense”, informed by the belief that too much physical activity threatens recovery, parallels Lim et al.’s study which reported that FCs tend to emphasize the importance of rest to their hospitalized relative’s recovery [7]. Likewise, Najjar et al. found that FCs were hesitant to have their relatives mobilize in hospital because of the potential health dangers [8].

Overall, our study underscores how resistant many FCs were to supporting their relative’s physical activity because doing so led to interpersonal conflict. Our finding that a lack of clear guidelines created space for patients to challenge their FC’s attempts at promoting physical activity echoes Mitchell et al.’s finding indicating that when FCs and patients are poorly prepared for discharge, they experience confusion which can give rise to conflict [25]. Such friction may be rooted in FC-patient dynamics and may exacerbate conflictual relationships which are often characterized by disagreements surrounding each other’s capabilities, level of involvement in care, and healthcare decision-making [26]. In a systematic review of studies examining how FCs and patients support each other during patients’ chronic and terminal health conditions, McCauley et al. found that shifts in power dynamics were associated with role reversal, particularly in instances where the FC was historically in a subordinate position [27]. It may be that FCs in our study were wary of such conflicts when they indicated that they did not want to take on the responsibility for their relative’s physical activity. It is also possible that healthcare provider support for patient physical activity after discharge may help mitigate conflicts between patients and their FCs; however, the feasibility of providing such support may be limited in rural communities given the wide geographical distances between patients and limited human healthcare resources. Lastly, it is conceivable that some FCs many never see this as their role and will remain unenthusiastic about supporting their relative’s physical activity.

### Implications for practice and policy

Our findings have several implications for practice. Healthcare providers should assess FCs’ receptivity to supporting their relative’s physical activity and can prepare FCs of patients at risk for hospital readmission by giving them information on their relative’s capacity for physical activity, how it promotes recovery, and how to support it. Healthcare providers should be conscious that FCs’ beliefs may lead to them suppressing their relative’s physical activity and should educate FCs on the hazards of inactivity in the post-discharge period. Ensuring that FCs recognize the importance of physical activity and promote it is essential because prior studies have identified that adults are more physically active when they are supported by family members [28]. Healthcare providers may consider modelling strategies that FCs can use to promote patient activity starting in hospital. Clear, rather than vague, physical activity guidelines are needed because the latter are open to interpretation and have the potential to serve as a source of friction. Healthcare providers should be attuned to the potential for FC-patient conflict when relying on FCs to support patients’ physical activity. Ideally, physical activity instructions should be given to FCs and patients together to provide the opportunity to seek clarification and achieve consensus about the activity plan moving forward.

On a policy level, decision-makers should note that our call for better FC preparation should not be construed as an endorsement of having FCs take on roles that are beyond their abilities. Overestimating FCs’ capacity to take on the seemingly straightforward task of supporting a relative’s physical activity could lead to an important element of post-discharge care being neglected or ignored altogether.

### Implications for research

In terms of implications for future research, more studies are needed on FCs’ perspectives of rest and physical activity in the post-discharge period, particularly in FCs of patients at risk for hospital readmission in rural settings. Studies targeting this population are urgently needed given that our findings demonstrate the multiplicity of constraints influencing FCs’ ability to support their relative’s physical activity. While our study identified that FCs reported receiving vague instructions from healthcare providers on how to support physical activity, future studies with healthcare providers are needed to explore the nature of their instructions surrounding physical activity. It is possible that healthcare providers attempted to put instructions in lay terms that are accessible to FCs but in doing so may have inadvertently rendered them imprecise and open to interpretation. Future research is also needed to explore how pervasive the belief is amongst FCs that rest should supersede physical activity during recovery, as well as whether and how such beliefs restrict patients’ physical activity. Lastly, more research is needed on interpersonal conflict rooted in the promotion of physical activity and how to address it. For example, studies should examine the precise nature of the conflict in the post-discharge period and the factors (e.g., lack of clear guidelines) influencing conflict and physical activity adherence.

## Limitations

The study was conducted in one province of Canada, which may limit the transferability of the findings to other rural jurisdictions. Our entire sample identified as Caucasian and thus, our findings may not speak to the full diversity of perspectives on challenges related to supporting the physical activity of a relative discharged from hospital.

## Conclusions

Despite assertions that FCs are a potential source of support for patient physical activity, FCs are largely unprepared to support the physical activity of a relative recently discharged from hospital. More work needs to be done to ensure FCs who are willing and able can assume that role and perform it effectively. This is particularly important given that many patients are discharged with newly acquired functional declines [22] and many rural communities have limited formal healthcare services to support them. FCs need information on their relative’s capacity for physical activity, how it promotes recovery, and clear guidelines on how to support it. Healthcare providers should be conscious of the potential for family caregiver-patient conflict surrounding physical activity, assess family caregivers’ ability and willingness to support physical activity, educate them on the hazards of inactivity, and provide physical activity instructions to family caregivers and patients conjointly.

## Supplementary Information

**Additional file 1.**

## Data Availability

According to the guidelines of our hospital site’s Research Ethics Board, we are not permitted to post the study data for public consumption. Given that our sample was recruited from small towns and participants narratives may be recognizable to others even in de-identified data, we are also unable to share the data by request.

## Abbreviation

FC: Family Caregiver
