# Supplementary material for: Family caregivers’ preparedness to support the physical activity of patients at risk for hospital readmission in rural communities: an interpretive descriptive study
Source: BMC Health Serv Res. 2022 Jul 13;22:907. doi: 10.1186/s12913-022-08289-4 (PMC9281041; doi:10.1186/s12913-022-08289-4)
Supplement: Supplementary file 1 — Additional file 1. [file 12913_2022_8289_MOESM1_ESM.docx]

**Appendix A**

**Semi-Structured Interview Guide for Families**

1. I would like to know about your experience since your relative came home from the hospital and some of the things that you found challenging or hard to manage. I would like you to think back to your recent experience and to tell me, during the first couple of weeks after leaving the hospital, were there any parts of your relative’s care and recovery at home that you found hard to manage?

[Probes: Were you able to manage whatever physical activity (e.g., walking) was recommended for your relative after coming home? If not, why? Was there anything about the recommendations you found difficult to manage at home? Can you think of any things that would have made it easier for you to manage your relative’s physical activity and meet the goals that were set with the nurses and other healthcare providers?

2) Tell me about the help you received at the hospital to prepare you to manage your relative’s care and recovery at home. How could the nurses and other healthcare providers have better prepared you to manage your relative’s care and recovery after returning home?

[Probes for preparation for management:

- In your opinion, what could they have done better to prepare you to manage your relative’s care and recovery at home?]

3) Tell me about the help you received or have been receiving from healthcare providers since your relative coming home from the hospital in managing their care and recovery at home.

[Probes for care from nurses and other healthcare providers since coming home:

- At any point since coming home, did you need more help in managing your relative’s care and recovery? (e.g., manage rest and physical activity?)
- How could you have been better supported in managing your relative’s care and recovery at home?]

4) Other needs: Are there any other things you have found difficult and unprepared for now that your relative is back home that you think are important?
